# Development of a Reference-Free Indirect Bridge Displacement Sensing System

**DOI:** 10.3390/s21165647

**Published:** 2021-08-21

**Authors:** Jongbin Won, Jong-Woong Park, Junyoung Park, Junsik Shin, Minyong Park

**Affiliations:** 1 Department of Civil and Environmental Engineering, Chung-Ang University, Dongjak, Seoul 06974, Korea; sac1721@cau.ac.kr (J.W.); pjy5451@cau.ac.kr (J.P.); jacoom1030@cau.ac.kr (J.S.); 2 Banseok Safety Cooperation, Namyangju-si 12014, Korea; miyon11@naver.com

**Keywords:** displacement measurement, reference-free displacement, wireless sensor, strain, acceleration

## Abstract

Bridge displacement measurements are important data for assessing the condition of a bridge. Measuring bridge displacement under moving vehicle loads is helpful for rating the load-carrying capacity and evaluating the structural health of a bridge. Displacements are conventionally measured using a linear variable differential transformer (LVDT), which needs stable reference points and thus prohibits the use of this method for measuring displacements for bridges crossing sea channels, large rivers, and highways. This paper proposes a reference-free indirect bridge displacement sensing system using a multichannel sensor board strain and accelerometer with a commercial wireless sensor platform (Xnode). The indirect displacement estimation method is then optimized for measuring the structural displacement. The performance of the developed system was experimentally evaluated on concrete- and steelbox girder bridges. In comparison with the reference LVDT data, the maximum displacement error for the proposed method was 2.17%. The proposed method was successfully applied to the displacement monitoring of a tall bridge (height = 20 m), which was very difficult to monitor using existing systems.

## 1. Introduction

Structural health monitoring (SHM) provides information about the current condition of a structure, thereby assisting decision-making for operation and maintenance. Continuous SHM allows early-stage damage detection, reduces the downtime, and prevents potential failure during operation. Vertical displacement induced due to traffic loading is regarded as a crucial measurement in bridge health monitoring because the displacement change is negligible under ambient traffic loads. Ambient displacement monitoring detects any structural damages or stiffness degradation of a bridge. Traditionally, the displacement is measured using a linear variable differential transformer (LVDT), a contact-type sensor that provides accurate displacements. However, the installation of LVDTs to bridges is limited, as they must be affixed on a stationary reference [1,2,3]. Therefore, LVDT measurements are limited to only some points on a structure and are unavailable to bridges built over waterways or highways. Recently, the computer vision-based method has emerged as an alternative to the traditional method as it accurately measures the structure using a camera installed on a remote stationary reference instead of that on a bridge [4,5,6,7]. However, as in the case for optical sensors, the accuracy of the computer vision-based method is highly affected by lighting conditions and wind or ground vibrations that move the camera. Alternatively, the light detection and ranging (LiDAR)-based system and radar-based displacement measurement systems have been introduced because of robustness to weather conditions [8,9,10,11,12]. However, these systems are vulnerable to self-motion; therefore, structural displacement measurements of a full-scale bridge are still challenging.

To address the limitations of reference-based methods requiring stationary reference for sensor installation, wireless smart sensors have been developed to measure the structural responses and to estimate displacement indirectly. Acceleration-based displacement estimation for reference-free measurement can be performed through direct integration in the frequency domain using finite impulse response (FIR) filters. Although FIR filters can eliminate the error from double integration of acceleration, only dynamic displacement can be reconstructed [13,14,15].

In addition to acceleration, strain measurements can be used to estimate reference-free displacement based on strain-displacement relationships [16,17,18]. Strain-based displacement methods can reconstruct pseudo-static displacement, but there is a lack of high-frequency components of displacement. Moreover, since the location of a neutral axis cannot be accurately determined, it is difficult to determine the magnitude of the displacement.

To overcome the issues of strain and acceleration, data-fusion-based, reference-free indirect displacement estimation was developed to estimate bridge displacements [19]. Reference-free indirect measurements can be useful for bridge ratings because they can easily estimate the flexural displacement of a bridge at any location. For instance, Park et al. [19] proposed the use of a wireless sensing system for the estimation of indirect displacement. A total of five wireless sensor nodes were distributed, each equipped with one strain channel and three acceleration channels [20,21]. The wireless sensors successfully acquired the distributed strain and acceleration measurements for the indirect displacement estimates, but acquiring multichannel data can be challenging in practical implementations because the bridge superstructure may interfere with the displacement signals [22,23].

This study develops a practical indirect displacement sensing system with multimetric sensing that simultaneously measures three-channel accelerations and three-channel strains. First, the multimetric board capable of simultaneously observing the three-axis acceleration and three-channel strains was proposed. Second, to enhance the accuracy of the displacement estimation, the placements of the three-channel strains were optimized in a numerical study. The indirect bridge displacement estimation method was optimized for three distributed strain measurements and one acceleration measurement. The proposed system was realized using a commercial wireless sensor platform called Xnode [24,25,26], and the proposed system’s performance was experimentally validated through full-scale bridge applications. The estimated displacements were compared with those of a reference LVDT.

The remainder of this paper is organized as follows. Section 2 describes the development of a multimetric sensor board and its integration onto the Xnode platform. Section 3 explains the indirect displacements derived from the strain and acceleration measurements and determines the optimal locations of the three strain sensors through a numerical study. In Section 4, the proposed method is experimentally validated on concrete- and steel-box girder bridges and the experimental results are compared with the reference measurements. The paper concludes with Section 5.

## 2. Development of a Multimetric Sensing Board

### 2.1. Design Challenges

A multimetric sensing of acceleration and strain is required for multiple and high-sensitivity vibration and strain sensing. The accelerometer should be carefully designed to capture the vibrations not only of lightly damped steel structures but also of highly damped concrete structures. Multiple small strain responses (<1 us) must be captured on the bridge surface. The wireless sensor platform on which the developed multimetric sensor board is integrated should be carefully selected. Therefore, when developing a multimetric sensor board for wireless sensors, the following three typical design challenges must be considered: (1) identifying an appropriate wireless sensor platform with high processing capability and low power consumption; (2) designing a board with a high-resolution accelerometer and strain sensor with power management; and (3) designing a stable and low-noise printed circuit board (PCB) for physically integrating the accelerometer with the wireless sensor platform.

### 2.2. Xnode

**Figure 1:**
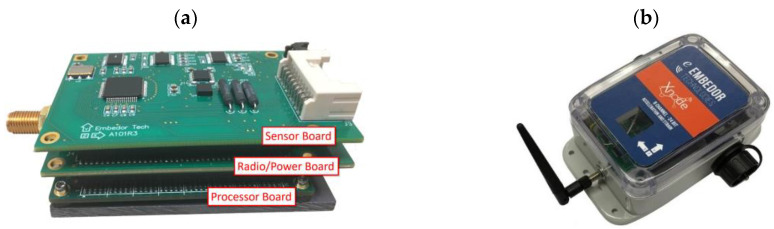
Xnode smart sensor: (**a**) three-board stack and (**b**) enclosure.

To measure multiple accelerations and strains at high sampling rates (e.g., 100 Hz), the base wireless sensor platform requires sufficient processing capability for implementing real-time digital filtering and recording. For the wireless sensor platform for multimetric sensing, we selected Xnode for its high processing speed and sufficient synchronous dynamic random access memory (SDRAM) size for real-time data processing. The standard Xnode consists of three main boards: the processor, radio/power, and sensor boards (Figure 1a). The processor board was a customized Mini4357 developed by Embest Technology (Shenzhen, China). It has an LPC4357 microprocessor [27] with a 204-MHz clock speed and 32 MB of SDRAM for temporary data storage and processing. Mini4357 has numerous interfaces, including general purpose input/output (GPIO) pins and peripheral interfaces, such as serial peripheral interfaces and an inter-integrated circuit.

The radio/processor board has a 2.4-GHz Zigbee radio for low-power wireless communication (Atmel AT86RF233) [28]. After adding a radio booster, the communication range reached beyond 1 km. An integrated circuit for charging and regulation of power was also added.

### 2.3. Multimetric Sensor Board

**Figure 2:**
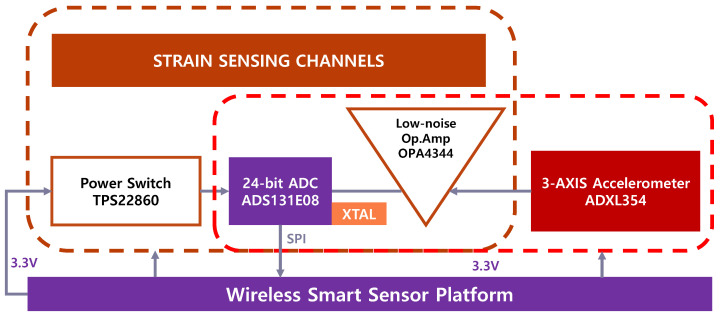
Configuration of the developed multimetric board.

The configuration of the developed multimetric board is shown in Figure 2. The board employs a 24-bit analog to digital converter (ADC) (ADS131E08, Texas Instruments [29]) for data acquisition. ADS131E08 is a delta–sigma ADC supporting eight differential inputs with a high sampling rate (up to 64 ksps). Three out of a total of eight channels were allocated to high-sensitivity three-axis acceleration measurements by ADXL354 [30], and three channels were integrated with a Wheatstone bridge for strain sensing. The two remaining channels were open for external analog voltage sensing. The three-axis accelerometer ADXL354 was selected for its low-noise power density of 20 μg√Hz. ADXL354 provides a low noise of 0.16 mg with a bandwidth of 50 Hz under the ±2 g sensing range. The ADXL354 was deployed apart from the ADC on the board design to minimize the temperature effect, otherwise, the ADC can experience an increase in heat due to the heat transferred from the ADXL354 at start-up.

**Figure 3:**
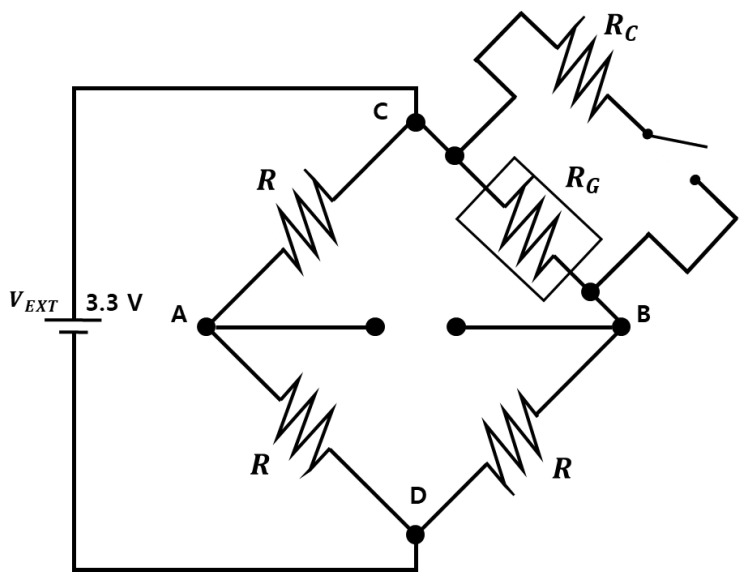
Wheatstone bridge.

A three-channel-strain sensing circuit was designed using a quarter-bridge, which is a type of Wheatstone bridge (Figure 3). $V_{EXT}$ is the input voltage, $R_{G}$ is the resistance of the strain gauge, and $V_{A}$ and $V_{B}$ are the voltages measured at points A and B, respectively. The calibration resistance $R_{C}$ was fixed at 100 kΩ. Depending on $R_{G}$, R could be switched between 120 and 350 Ω using a switch.

The voltage difference between points A and B was calculated as

(1) $$\Delta V=\frac{GF\varepsilon }{4}V_{EXT}=\frac{\Delta R_{G}}{4R}V_{EXT},$$

where GF is the gauge factor and *ε* is the strain. The resistance change in the strain gauge is linearly related to the voltage difference. Using Equation (1) with GF = 2, the voltage corresponding to a strain change of 1 μs was obtained as  1.65 μV. In the 24-bit ADC, the effective number of bits was 18 and the programmable gain was 12; therefore, the resolution of the ADC was calculated as 1.02 μV, affirming that the ADC can measure within 1 μs. Besides the default strain sensing, strain calibration was enabled by a shunt-sensing circuit. Shunt calibration finds the conversion factor between the measured voltages and their corresponding strain values. The shunt can be calibrated by connecting a known resistance to the strain gauge in parallel, calculating the theoretical strain change, and measuring the corresponding voltage difference. Figure 3 is a circuit diagram of the shunt calibration. In this figure, $R_{C}$ denotes the known resistance for calibration. When the calibration resistor $R_{C}$ is connected with $R_{G}$, the resistance changes as follows:

(2) $$\Delta R_{G}=\frac{R_{G}R_{C}}{R_{G}+R_{C}}-R_{G}.$$

Based on the relationship between the GF and the resistance change given by Equation (3), we can compute the corresponding strain change:

(3) $$GF=\frac{\Delta R_{G}}{\epsilon R_{G}}.$$

Comparing the actual voltage difference before and after connecting the known shunt resistance, we obtain the conversion factor between the voltage and strain units.

Note that the half-bridge can be configured by replacement resistance between C and D in Figure 3 with a dummy gauge for temperature compensation to avoid self-heating of a strain gauge. The dummy gauge can be installed along an unstrained direction perpendicular to the active strain gauge to cancel out temperature effects.

To handle the limited power resources available on the wireless sensor, a power management circuit was developed to control the power on demand. The most power-consuming part is strain sensing, where one 120-Ω strain gauge draws $V_{EXT}/2R=13.8\;\mathrm{mA}\;\mathrm{of}\;\mathrm{current};\;\mathrm{thus},\;$ three strain gauges consume 41.3 mA. The power on the multimetric sensor board was controlled by a digital power switch (TPS22860) using GPIO on the wireless sensor platform. To minimize the power operation, the multimetric sensor board was maintained in deep sleep mode until triggered.

**Figure 4:**
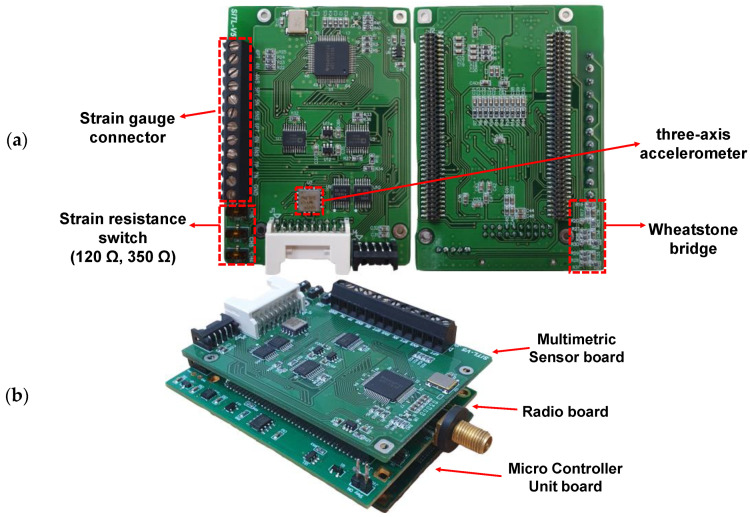
(**a**) Developed multimetric sensor board and (**b**) its integration with Xnode.

Figure 4 shows the developed multimetric sensor board which has an onboard strain gauge connector and three-axis accelerometers on the top side, and a Wheatstone bridge on the bottom side.

**Figure 5:**
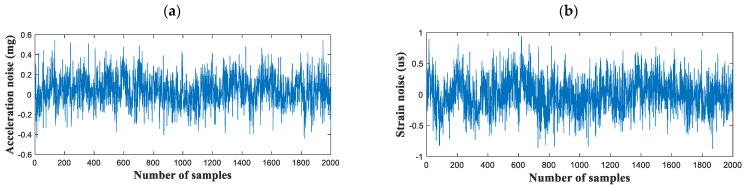
Noise level of the developed multimetric board: (**a**) acceleration and (**b**) strain.

The noise level of the developed multimetric board was validated via a laboratory-scale evaluation. This test confirmed whether the PCB met the specified accelerometer and strain performances. The resistance and GF of the strain gauge were 120 Ω and 2.1, respectively. A strain gauge was attached at the top of a 1.5-high cantilever beam. The integrated multimetric sensor board with Xnode was deployed at the support of the beam and measured at a sampling rate of 100 Hz. The conversion factor obtained in the shunt calibration was 48,287 μs/V. The acceleration and strain noise levels were measured over 20 s in a vibration-free environment, and the results are plotted in Figure 5. The standard deviations of the noises related to acceleration and strain were 0.15 mg and 0.29 μs, respectively. The acceleration noise was very similar to the datasheet value, indicating that the PCB was well designed and introduced no further signal noise. Meanwhile, the noise in the strain measurement was <1 μs with a programmable gain of 12.

## 3. Optimal Sensor Location for Reference-Free Indirect Displacement Estimation

This section estimates the performance of the reference-free indirect displacement from the strain and acceleration measurements, and investigates the optimal sensor location for the three strain measurements available on the multimetric sensor board through numerical analysis.

### 3.1. Reference-Free Indirect Displacement Estimation

The displacement can be indirectly estimated from the vertical acceleration and three-channel strains measured using the developed multimetric board. Park et al. [19] proposed a multimetric data fusion method for displacement estimation. The indirect displacement estimation method is based on the following relationship between strain and displacement:

(4) $$u_{strain}=\Phi q=\Phi \Psi \varepsilon =\alpha \Phi \Phi \prime \prime \varepsilon .$$

where $u_{strain}$ is the strain-based displacement, Φ and Ψ are the displacement- and strain-mode shape matrices, respectively, and α is the scaling factor, which critically determines the strain–displacement relationship. α can be obtained using both acceleration- and strain-based displacements from Equation (4) by matching the magnitude of power spectral density of the strain-based displacement to the acceleration-based displacement (obtained by double integration of the acceleration):

(5) $$\alpha =\sqrt{\frac{S_{strain,xi}^{disp}\left(f_{n}\right)}{S_{acc,xi}^{disp}\left(f_{n}\right)}}.$$

Here, $S_{strain,xi}^{disp}$ and $S_{acc,xi}^{disp}$ are the power spectral densities of the strain- and acceleration-based displacements at *x_i._*, respectively. $f_{n}$ is the most dominant natural frequency, which is generally the first natural frequency of the structure.

### 3.2. Setup for Numerical Analysis

A numerical study was conducted on the locations of the three strain measurements available for the developed multimetric sensor board. The sensor deployment was symmetric about the center, as suggested by Park et al. [19]. Specifically, one of the strain gauges was fixed at the center of the bridge and the other strain gauges were deployed equidistantly from the center. However, the length of the bridge is very diverse, and it was necessary to determine the optimal installation position according to the span length of the bridge. In addition, the greater the distance from the center, the more difficult it is to install the gauge, so the optimal location with a minimum distance had to be determined. In this section, the effect of strain-gauge distance from the center was evaluated on bridges of different lengths.

Simulations were performed on simply supported beams of various lengths. The cross section of the model was 2 m × 1 m (width × height), the modulus of elasticity was 210 GPa, the density was 7850 $\mathrm{kg}/m^{3}$, and the damping ratio was 5%. The structure was excited by a traffic load comprising a two-wheel car and a three-wheel truck. For the two-wheel car, the force on both the wheels was 20 kN and a 10% random force was applied to the front and rear axles, which were separated by 2.9 m. For the three-wheel truck, the force values were 35 kN with a 10% random force on the front axle and 145 kN with a 10% random force on both of the rear axles. The front and rear axles were separated by 4.3 m, and the separation of the two rear axles was arbitrarily adjusted in the range of 1–9 m, imposing randomness in the moving load. The vehicle configurations in the numerical simulation are given in Table 1.

The simply supported beam was modeled using an *n*-element Bernoulli beam, where *n* equals the length of the beam. For instance, a 30–m long beam comprised 30 elements and 29 nodes for simulating the acceleration, strain, and displacement. The estimated displacement in the proposed method was derived by simulating the vertical acceleration of the beam center and the three strain channels. The estimated displacement was then compared with the exact displacement obtained in the simulation. Three cases with different beam lengths (30, 40, and 50 m) were simulated using symmetric strain-gauge deployments. One gauge was located at the center of the bridge while the other gauges were deployed equidistant from the center. In the simulation, the distance between the center and the two side gauges was manipulated, and the estimated displacement was compared with the exact displacement. For instance, along the 30-m long beam, the off-center gauges were located at 1–14 m from the center. At each strain measurement location, the acceleration and strains were simulated 100 times under various moving loads and the displacement was estimated. The error was defined as the root mean square error (RMSE) of the difference between the estimated and exact displacement divided by the maximum displacement (Equation (6)). The errors in the 100 simulations were averaged for comparison.

(6) $$\mathrm{Error}=\frac{\sigma \left(u_{estimated}-u_{exact}\right)}{\max\left(\left|u_{exact}\right|\right)}.$$

where $u_{estimated}$ and $u_{exact}$ are estimated and exact displacement, respectively.

### 3.3. Results and Discussion

**Figure 6:**
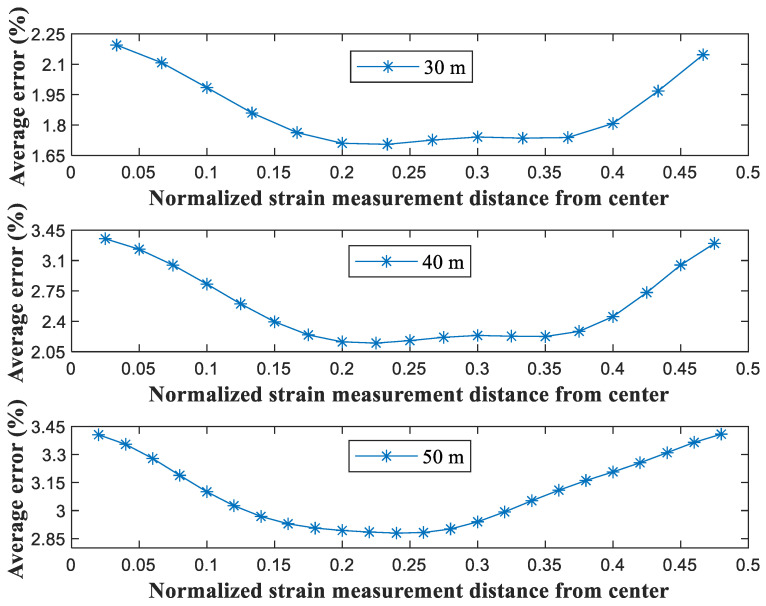
RMSE error dependence on the normalized distance from the center of the bridge.

Figure 6 shows the average error in the strain measurements as the location changed from 0 to 0.5 × *L* from the center, where *L* is the bridge length. The optimal location of the strain measurements (normalized by the bridge length) was 0.2–0.25 × *L* from the center. For example, on the 30-m long bridge, the optimal position was 0.2 × 30 m (i.e., 6 m) from the center. Interestingly, the measurements of the location of the strain from the center did not critically affect the error (the average error increment was 1–2%). At the sites where sensor installation was difficult, the strain sensors were located close to the center for the estimation of displacement.

## 4. Experimental Validation

### 4.1. Validation on the Keukrak Bridge

**Figure 7:**
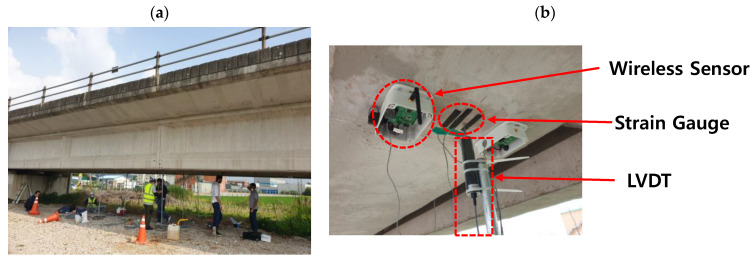
Experimental setup: (**a**) overview and (**b**) sensor deployment.

The proposed reference-free indirect displacement sensing system was experimentally validated on a prestressed concrete bridge located at Gwangju-si, South Korea. The experimental setup is presented in Figure 7. The integrated wireless sensor was installed at the mid span of the beam, which was 21-m long, and two strain gauges were attached 4 m from the either side of the center, which is 0.2 of the span length (Table 2). The results of the strain sensing system installed at the mid-span were compared with those of an LVDT installed at the same location.

**Figure 8:**
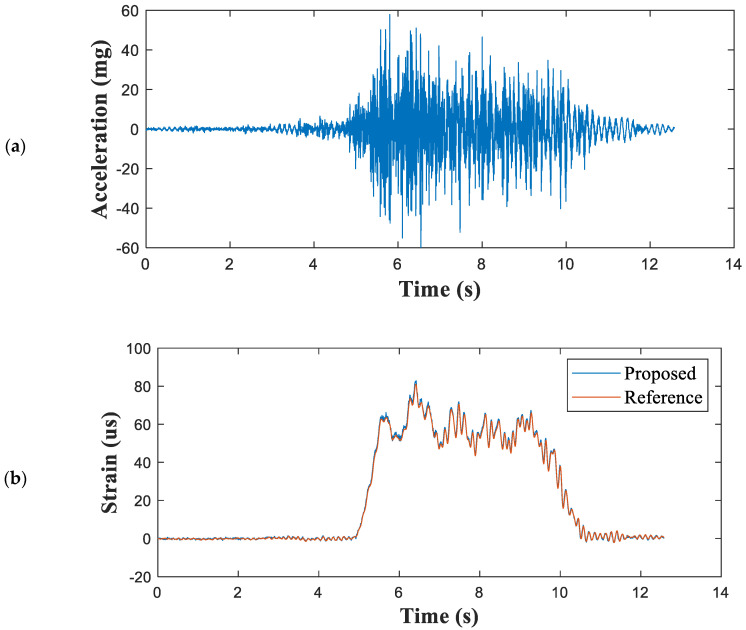
Measured structural responses of the Keukrak Bridge: (**a**) vertical acceleration and (**b**) strains at mid-span point.

**Figure 9:**
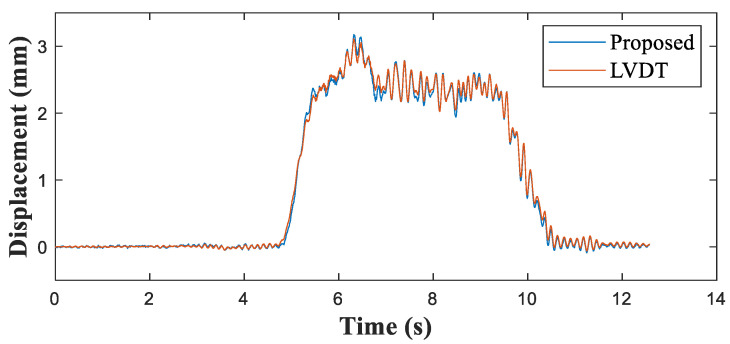
Comparison of structural displacements at the mid-span point of the Keukrak Bridge.

Figure 8 shows the measured acceleration and a comparison of the measured strain and reference signals. The strain of the mid-span point measured by the developed sensor aligned very well with the reference strain measurements (Figure 8b). Figure 9 compares the displacements estimated using the proposed method and the LVDT. Table 3 presents the maximum peak displacements obtained by the proposed method and the reference. The error in the displacements of the proposed method relative to the LVDT displacements was no greater than 2.17%.

### 4.2. Validation on the Jojungchun Bridge

**Figure 10:**
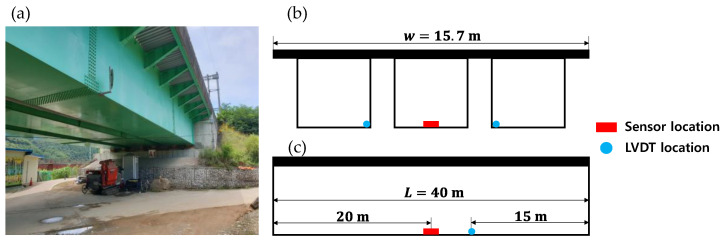
Experimental setup showing (**a**) the steel-box girder bridge and (**b**) its sectional and (**c**) lateral views.

A second experiment was performed on a 40-m long steel-box girder bridge located at Gapyeong-gun, South Korea. An overview of the experimental setup is presented in Figure 10. Two reference LVDTs were installed at the endpoint of each side cell (rather than the middle cell) to monitor the bidirectional passage of trains (Figure 10b). An integrated wireless sensor was installed at the center of the box. As this bridge is a continuous bridge with a simple support at one end, its displacement should be at its maximum at the 3/8 point (i.e., at 15 m). Hence, the LVDT was installed 15 m from the simply supported end. The displacement at the same point was indirectly estimated, and the reference displacement at the sensor location was interpolated between the two LVDT displacements for comparison. Table 4 gives the configurations of the experimental validation.

**Figure 11:**
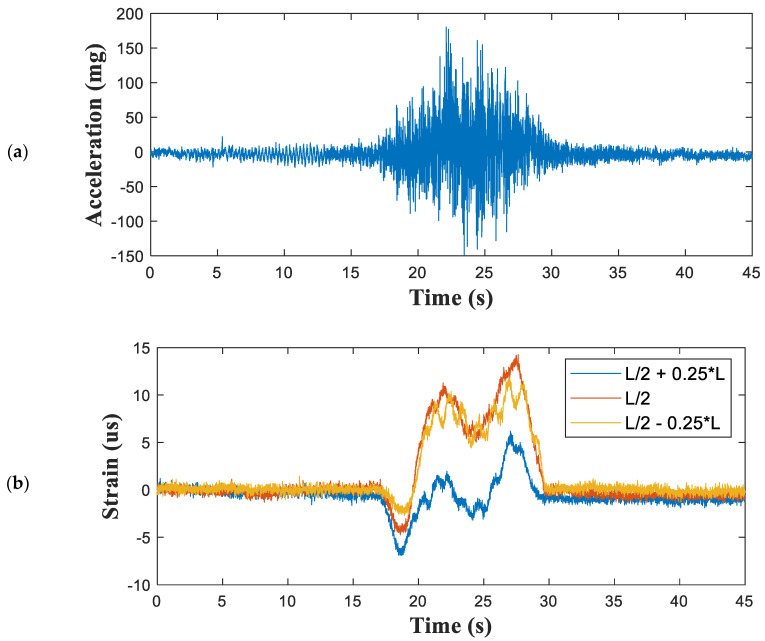
Measured structural responses from the Jojungchun Bridge: (**a**) vertical acceleration and (**b**) strains at three points.

**Figure 12:**
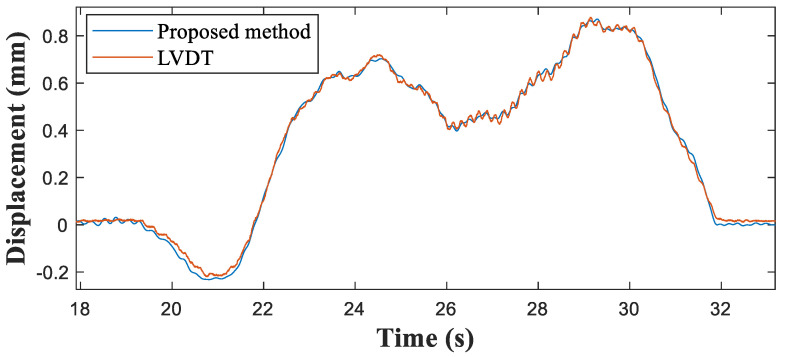
Comparison of displacements at the 3/8 span point of the Jojungchun Bridge.

Figure 11 presents the vertical acceleration at the mid-span point and the strains measured at three points where the strain measurements were collected at a distance of 10 m from the simply supported end (at 0.25 of the span length). The strain responses were asymmetric because the boundary conditions differed at each end. One end was a simple support while the other was continuous. Figure 12 compares the reference and indirect displacements estimated by the proposed sensing system. The maximum displacement estimated by the proposed method was 0.8795 mm. Compared with the 0.8780 mm estimated by the reference data, this gave a maximum displacement error of 0.17% (Table 5).

### 4.3. Application of Developed System to the Geumgok Bridge

**Figure 13:**
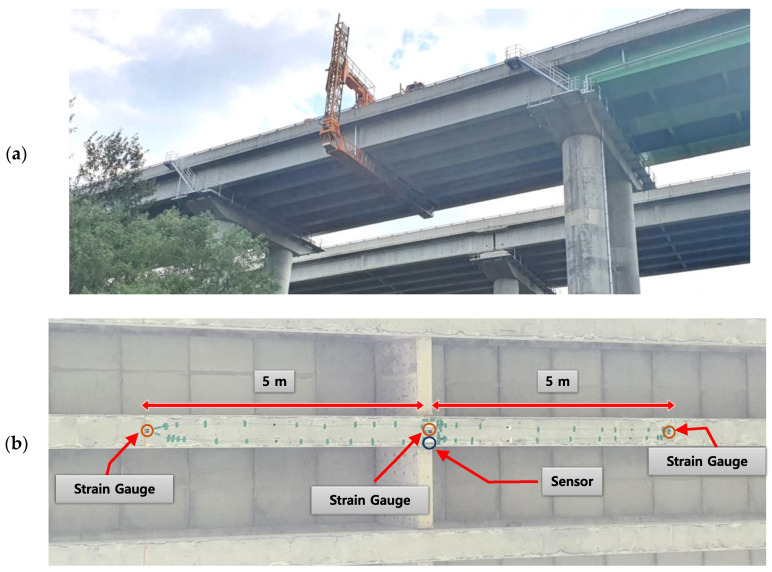
Experimental setup: (**a**) the Geumgok Bridge; (**b**) strain-gauge locations.

To demonstrate the efficiency of the proposed cloud-based monitoring system, the developed sensor was instrumented on a tall (20-m high) concrete bridge located at Yeongdong-gun, South Korea. As the proposed indirect displacement sensing system is applicable to any type of bridge, the Geumkok Bridge was selected as the testbed because, to the best of our knowledge, its displacement has not been previously monitored. The developed sensor was installed beneath the bridge deck, where it could detect ambient vibrations. The sensor and strain gauges were installed and instrumented by a bridge inspection truck (Figure 13). The span of the bridge was 35 m and the strain measurements were collected at a distance of 5 m from the center (0.15 of the span length).

**Figure 14:**
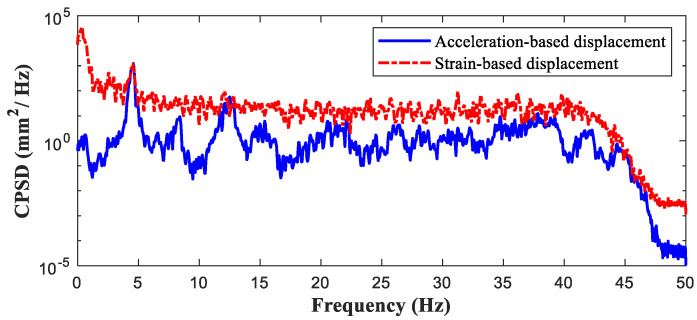
Scaled cross power spectral densities (CPSDs) of acceleration and strain on the Geumgok Bridge.

The scaling factor was obtained from the strain and acceleration measurements using Equation (5). Figure 14 plots the cross power spectral densities (CPSDs) of the acceleration- and strain-based displacements. The scaling factor was 0.044 at the first natural frequency of 4.49 Hz. At this frequency, the CPSD of the calibrated strain-based displacement exhibited the same power as the acceleration-based displacement.

**Figure 15:**
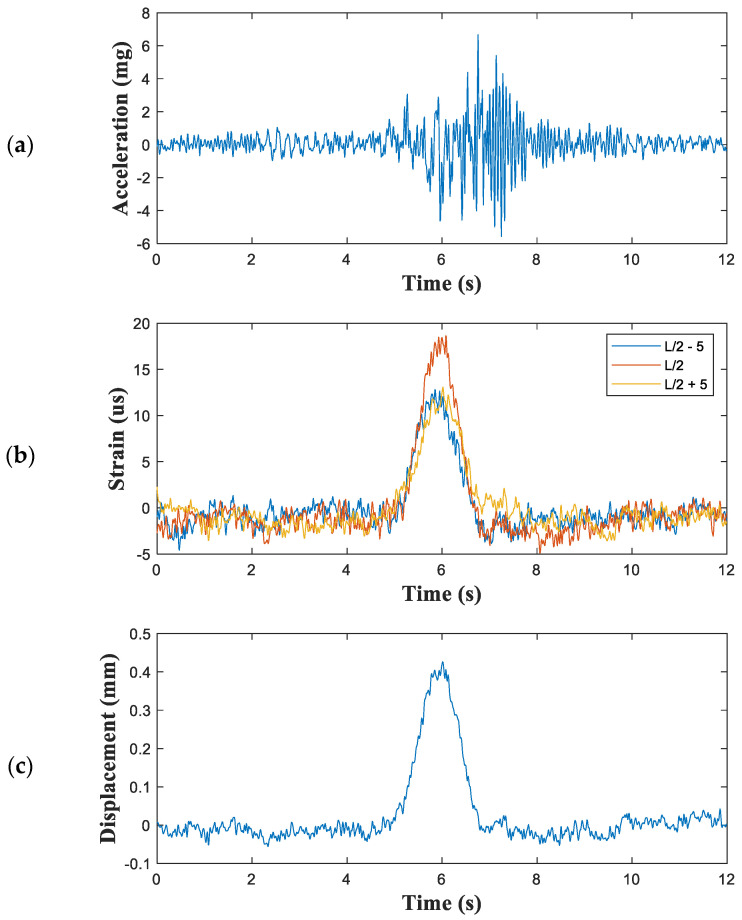
Measured structural responses and estimated displacements of the Geumgok Bridge: (**a**) measured vertical acceleration; (**b**) three-channel strains; and (**c**) estimated displacements.

**Figure 16:**
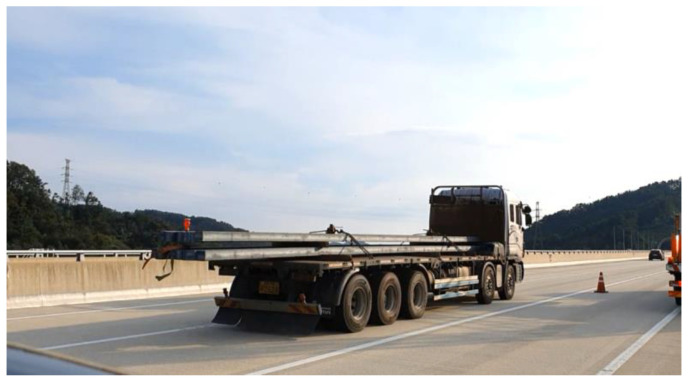
Five-wheel heavy truck crossing the Geumgok Bridge.

The structural responses with unknown input were monitored via video recordings during the measurements. Figure 15 shows an instance of the measured vertical acceleration and three channel strains as well as the estimated displacement caused by a five-wheel truck carrying steel plates (Figure 16). The developed system captured displacements as small as 0.1 mm from the set of strain and acceleration measurements, and the maximum displacement was 0.42 mm.

## 5. Conclusions

In this paper, we developed and evaluated a reference-free indirect displacement sensing system. The system integrates a multimetric board that measures three strains and the three-axis acceleration using a commercial wireless smart sensor. The indirect displacement estimation method was optimized for three strain measurements and one acceleration measurement. In actual bridge monitoring, the number of wireless signals are inevitably reduced and the communication among multiple wireless sensors becomes difficult. For this reason, the proposed sensing system uses a single sensor unit for reference-free bridge displacement estimation.

The main contributions of this study are as follows:

- The developed multimetric board simultaneously observed the three-axis acceleration and three-channel strains and estimated the reference-free displacement.
- To enhance the accuracy of the displacement estimation, the placements of the three-channel strains were optimized in a numerical study.

When developing the multimetric sensor board for wireless sensors, the following design challenges were addressed: (1) the board required a high-resolution accelerometer and strain sensor with power management; (2) the board was integrated with three Wheatstone bridges for three-channel strain sensing; and (3) an automated shunt calibration was developed and implemented in the software. Furthermore, the optimal location of strain sensing for the estimation of indirect displacement (0.2–0.25 of the span length) was numerically validated. For validation purposes, the developed multimetric board was integrated with the commercial wireless sensor platform Xnode. The integrated system was experimentally validated on two differently constructed railroad bridges and the estimated displacements were compared with those of LVDTs. On the Keukrak and Jojungchun Bridges, the maximum displacement errors were 2.17% and 0.17%, respectively. Finally, the developed system was applied to a tall (20-m high) bridgewhich was very difficult to monitor by using existing systems. The indirect displacements were successfully obtained, confirming that the proposed system can capture very small (<0.1 mm) reference-free displacements.

Future work is underway to develop ambient bridge displacement monitoring by expanding the proposed sensing system to cloud-based bridge management, where sensor data is uploaded in a cloud database and data processing for displacement estimation can be implemented in an autonomous manner.

## Figures and Tables

**Table 1:** Configurations of the moving load in the simulation.

Configurations	Front	Rear	
Two-wheel car	20 kN	20 kN	
Three-wheel truck	35 kN	145 kN	145 kN

**Table 2:** Configurations of the validation experiment on the Keukrak Bridge.

Bridge Type	Span	Strain Measurement Distance from Center
Prestressed concrete	21 m	4 m

**Table 3:** Comparison of displacement measurements on the Keukrak Bridge.

Method	Maximum Displacement (mm)	Error (mm)	Error (%)
Proposed method	3.18	0.07	2.17
Reference displacement	3.11	-	-

**Table 4:** Configurations for experimental validation on the Jojungchun Bridge.

Bridge Type	Span	Strain Measurement Distance from Center
Three-cell steel-box girder	40 m	10 m

**Table 5:** Comparison of displacement measurements on the Jojungchun Bridge.

Method	Maximum Displacement (mm)	Error (mm)	Error (%)
Proposed method	0.8795	0.0015	0.17
Reference displacement	0.8780	-	-

## Data Availability

Not applicable.
